# Inhibition of Aβ Aggregation by Cholesterol-End-Modified PEG Vesicles and Micelles

**DOI:** 10.3390/pharmaceutics17010001

**Published:** 2024-12-24

**Authors:** Shota Watanabe, Motoki Ueda, Shoichiro Asayama

**Affiliations:** Department of Applied Chemistry, Tokyo Metropolitan University, Tokyo 192-0397, Japan; watanabe-shouta1@ed.tmu.ac.jp (S.W.); ueda-motoki@tmu.ac.jp (M.U.)

**Keywords:** Alzheimer’s disease, Aβ aggregation, drug delivery carrier, micelle, vesicle, cholesterol-end-modified PEG (Chol-PEG)

## Abstract

**Background/Objectives**: This study aimed to design and evaluate Chol-PEG_2000_ micelles and Chol-PEG_500_ vesicles as drug delivery system (DDS) carriers and inhibitors of amyloid-β (Aβ) aggregation, a key factor in Alzheimer’s disease (AD). **Methods**: The physical properties of Chol-PEG assemblies were characterized using dynamic light scattering (DLS), electrophoretic light scattering (ELS), and transmission electron microscopy (TEM). Inhibitory effects on Aβ aggregation were assessed via thioflavin T (ThT) assay, circular dichroism (CD) spectroscopy, and native polyacrylamide gel electrophoresis (native-PAGE). **Results**: Chol-PEG_2000_ micelles and Chol-PEG_500_ vesicles were found to exhibit diameters of 20–30 nm and 70–80 nm, respectively, with neutral surface charges and those physical properties indicated the high affinity for Aβ. At a 10-fold molar ratio, thioflavin T (ThT) assay revealed that Chol-PEG_2000_ delayed Aβ fibril elongation by 20 hours, while Chol-PEG_500_ delayed it by 40 hours against Aβ peptide. At a 50-fold molar ratio, both Chol-PEG_2000_ and Chol-PEG_500_ significantly inhibited Aβ aggregation, as indicated by minimal fluorescence intensity increases over 48 hours. CD spectroscopy indicated that Aβ maintained its random coil structure in the presence of Chol-PEG assemblies at a 50-fold molar ratio. Native-PAGE analysis demonstrated a retardation in Aβ migration immediately after mixing with Chol-PEG assemblies, suggesting complex formation. However, this retardation disappeared within 5 min, implying rapid dissociation of the complexes. **Conclusions**: This study demonstrated that Chol-PEG_500_ vesicles more effectively inhibit Aβ aggregation than Chol-PEG_2000_ micelles. Chol-PEG assemblies perform as DDS carriers to be capable of inhibiting Aβ aggregation. Chol-PEG assemblies can deliver additional therapeutics targeting other aspects of AD pathology. This dual-function platform shows promise as both a DDS carrier and a therapeutic agent, potentially contributing to a fundamental cure for AD.

## 1. Introduction

In the brains of Alzheimer’s disease (AD) patients, amyloid-beta (Aβ) peptides are produced by sequential proteolytic cleavages of a membrane-bound protein called amyloid-beta precursor protein (APP) by proteases known as β-secretase and γ-secretase [1,2]. These Aβ peptides, consisting of around 40 amino acid residues, are known for their high propensity to form β-sheets and aggregate. Aggregated Aβ peptides have been reported in numerous studies to form amyloid plaques, which induce neuronal cell death [3,4]. The neuronal cell death leads to brain atrophy and cognitive impairment, ultimately resulting in the onset of AD [5,6]. Such diseases triggered by conformational changes in peptides or proteins are collectively referred to as “conformational diseases” [7]. Therefore, controlling peptide conformation is crucial for the treatment of AD and other conformational diseases. Consequently, significant efforts have been made to develop AD therapeutics targeting Aβ. Various agents, such as secretase inhibitors, anti-Aβ peptides, and anti-Aβ antibodies have been developed [8,9]. Recently, antibody drugs named aducanumab, lecanemab, and donanemab have been approved by regulatory authorities [10]; however, issues such as adverse effects, including ARIA (amyloid-related imaging abnormalities), remain unresolved, and the therapeutic effects are limited [11]. The low transfer rate of conventional antibody drugs to the brain, approximately 0.1% of the serum antibody concentration, is considered to be a significant factor limiting their therapeutic efficacy [12]. To facilitate drug delivery to the brain, it is essential for the drug to cross the blood-brain barrier (BBB). The BBB plays a critical role in protecting the central nervous system (CNS) from potentially harmful blood-borne substances; however, it selectively permits the passage of certain molecules, presenting a major obstacle to effective brain-targeted drug delivery [13]. Recently, intranasal administration has gained attention as a non-invasive method for drug delivery that bypasses the BBB [14,15]. Although the precise mechanisms remain incompletely understood, studies have shown that drugs administered via the intranasal route can reach the brain through transport via the olfactory and trigeminal nerves [16]. Additionally, the high cost of antibody drugs makes widespread application among the large population of AD patients impractical.

The alteration in cholesterol levels in the brains of AD patients has been reported [17,18,19,20], suggesting a potential link between cholesterol metabolism disorders and AD. Although the brain constitutes only 2% of body weight, it contains approximately one quarter of the body’s total cholesterol [21]. Furthermore, numerous studies have demonstrated that Aβ interacts with lipid membranes, with a particular preference for binding to cholesterol-containing lipid membranes [21,22]. C. Duyckaerts’s research group observed the binding of cholesterol and Aβ in AD senile plaques [23], J. Fantini and co-workers have reported that cholesterol in a lipid membrane strongly bound to Aβ peptide [24], and J. R. Harris has confirmed that micelles of Chol-PEG also interact with Aβ peptides and fibrils by TEM observation [25]. However, whether such an interaction between Aβ and cholesterol inserted into lipid membranes inhibits or promotes the aggregation of Aβ is still under discussion due to the complex interplay of various factors, including cholesterol content, surface charge, and membrane fluidity [26,27].

So far, our laboratory has developed a cholesterol-end-modified PEG, as a drug delivery carrier and bioinert surface coating, which was found to spontaneously form micelles and vesicles (assemblies) in water [28,29]. We successfully obtained not only micelles but also vesicles from cholesterol-end-modified PEG with molecular weights of 2000 and 500, respectively. These assemblies involving cholesterol, which can bind to Aβ, are expected to be used not only as a drug carrier but also as an inhibitor of Aβ aggregation. Additionally, it has been reported that PEG-modified nanoparticles, such as these assemblies, enhance the efficiency of brain delivery via intranasal administration [30,31]. Although the optimal characteristics of nanocarriers for intranasal administration have yet to be fully elucidated, PEGylated nanoparticles (NPs) with a diameter of less than 200 nm and a surface charge close to neutral have been employed for efficient drug delivery and may be particularly suitable for intranasal administration [30,31,32].

Here, we investigated an inhibitory effect of cholesterol-end-modified PEG assemblies on aggregation of Aβ peptides, particularly Aβ_40_, which consists of 40 amino acids. It is well known that Aβ_42_, consisting of 42 amino acids, has a higher propensity for aggregation compared to Aβ_40_ [33], while it is possible that such an Aβ_42_ aggregation can be controlled by a decrease in Aβ_40_ aggregation [34]. In this study, we examined the effects on Aβ_40_ aggregation, because Aβ_40_ is a good therapeutic target to be produced at nearly ten times the rate of Aβ_42_ [35].

## 2. Materials and Methods

### 2.1. Materials

mPEG-NH_2_ (molecular weight of 2000, SUNBRIGHT^®^ ME-020EA) was purchased from NOF Corporation (Tokyo, Japan). mPEG-NH_2_ (molecular weight of 500, 767565) and Thioflavin T (ThT) were purchased from Sigma-Aldrich Co. LLC (St. Louis, MO, USA). Cholesterol chloroformate was purchased from TCI Co., Ltd. (Tokyo, Japan). Amyloid β-Protein (Human, 1–40) [HCl Form] (Aβ_40_) was purchased from PEPTIDE INSTITUTE, INC. (Osaka, Japan). All other chemicals of a special grade were used without further purification.

### 2.2. Preparation of Chol-PEG Assemblies

Chol-PEG assemblies (Chol-PEG_2000_ micelles and Chol-PEG_500_ vesicles) were self-assembled solely by dissolving them in potassium phosphate buffer, without stirring or ultrasonication.

### 2.3. Particle Size and Zeta Potential Measurement

A dynamic light scattering (DLS) method by an electrophoresis light-scattering spectrophotometer (ELS-Z2, Otsuka Electronics Co., Ltd., Tokyo, Japan) determined the size of the Chol-PEG assembles with each concentration in potassium phosphate buffer at room temperature. The zeta potential of the resulting sample was measured at room temperature by ELS with electrodes.

### 2.4. Transmission Electron Microscopy (TEM) Observation

Chol-PEG_2000_ and Chol-PEG_500_ assemblies were prepared in potassium phosphate buffer to 9.4 μmol and used as sample solutions for TEM observation. A TEM grid (Nisshin EM Co., Tokyo, Japan) was dipped into the sample solution for a few seconds. The excess solution was blotted away by filter paper. The sample was stained by 2% phosphotungstic acid solution. The samples on grids were observed by a JEM-1400 (JEOL Ltd., Tokyo, Japan) at an acceleration voltage of 120 kV. Chol-PEG_500_, prepared in deionized water at a concentration of 5.5 mM, was examined using a JEM-2100F (JEOL Ltd., Tokyo, Japan) electron microscope at an acceleration voltage of 200 kV with uranyl acetate as the staining agent.

### 2.5. Thioflavin T (ThT) Assay

Aβ_40_ was dissolved in a 0.1% aqueous ammonia solution to a concentration of 4.7 × 10^2^ μM. For each well of a 96-well black PP plate (Greiner Bio-One, Co., Ltd., Frickenhausen, Germany), 2 μL of this solution was used. ThT was mixed to a final concentration of 9.4 × 10^−3^ μM, and Chol-PEGs were added at molar ratios of 10 and 50 relative to Aβ_40_. The total volume was adjusted to 100 μL per well. Each sample was prepared in triplicate (*n* = 3). As controls, NH_2_-mPEGs with the same molar concentrations as Chol-PEGs were prepared for each PEG molecular weight. Fluorescence intensities were measured every 15 min while incubating the prepared samples at 37 °C using a SpectraMax^TM^ mini (Molecular Devices LLC., US). A pre-shake was set for 60 s before each measurement. The excitation wavelength was 485 nm, and the emission wavelength was 535 nm. The dissociation of Aβ_40_ aggregation was evaluated under the same concentration conditions as the ThT assay described above. A sample containing Aβ_40_ alone was prepared following the same procedure as described previously, and aggregation was induced at 37 °C for 72 h. Subsequently, Chol-PEG_500_ was added at a molar ratio of 50 relative to Aβ_40_, and the sample was incubated again at 37 °C. Fluorescence intensity was measured hourly during the incubation.

### 2.6. Circular Dichroism (CD) Measurement

All samples were adjusted to the same concentration as in the ThT assay. The CD spectra from 200 nm to 250 nm of the resulting sample were measured with a J-820 spectropolarimeter (JASCO Corporation, Tokyo, Japan).

### 2.7. Naive Polyacrylamide Gel Electrophoresis (Native-PAGE)

The preparation of Aβ_40_ and Chol-PEGs was adjusted so that the final concentration and total amount matched those used in the ThT assay. The prepared samples were incubated at 37 °C, and gel electrophoresis was performed at 0 and 72 h. A 7.2 μL aliquot from each sample was taken and mixed with 0.8 μL of 10× loading buffer, then loaded onto an 8% polyacrylamide gel. The prepared gel was run using a buffer (pH 8.3) comprising 50 mM tris (hydroxymethyl)aminomethane (Tris) and 38 mM glycine. Electrophoresis was performed at room temperature for 10 min, and the electric current was maintained at 24 W using a WSE-1010 Compact PAGE Ace (ATTO Co., Tokyo, Japan). After electrophoresis, the gel was shaken in a fixing solution (methanol: water: acetic acid = 4: 5: 1) for 30 min, followed by staining with Coomassie brilliant blue (CBB) for 30 min. The gel was then imaged using a GelDoc Go Imaging System (Bio-Rad Laboratories Inc., Hercules, CA, USA). The captured images were adjusted for brightness (+40%) using Microsoft PowerPoint.

## 3. Results

### 3.1. Physical Properties of Chol-PEG Assemblies

**Figure 1:**
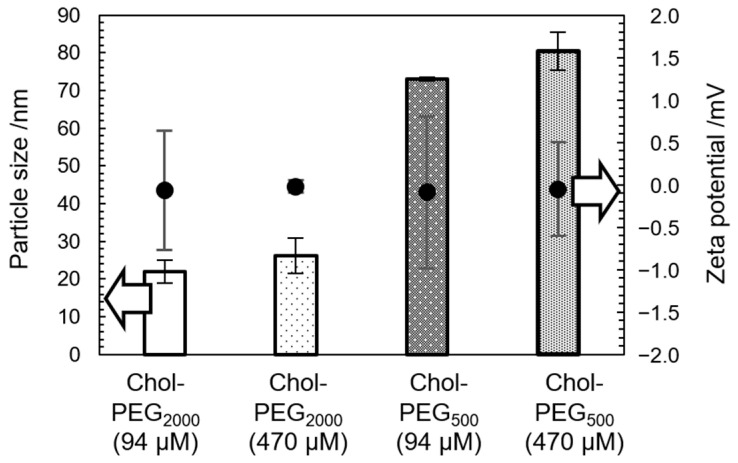
Particle sizes and zeta potentials of Chol-PEGs assemblies at each concentration used in later Aβ experiments. All concentrations of Chol-PEGs assemblies were above the critical aggregation concentration (CAC).

**Figure 2:**
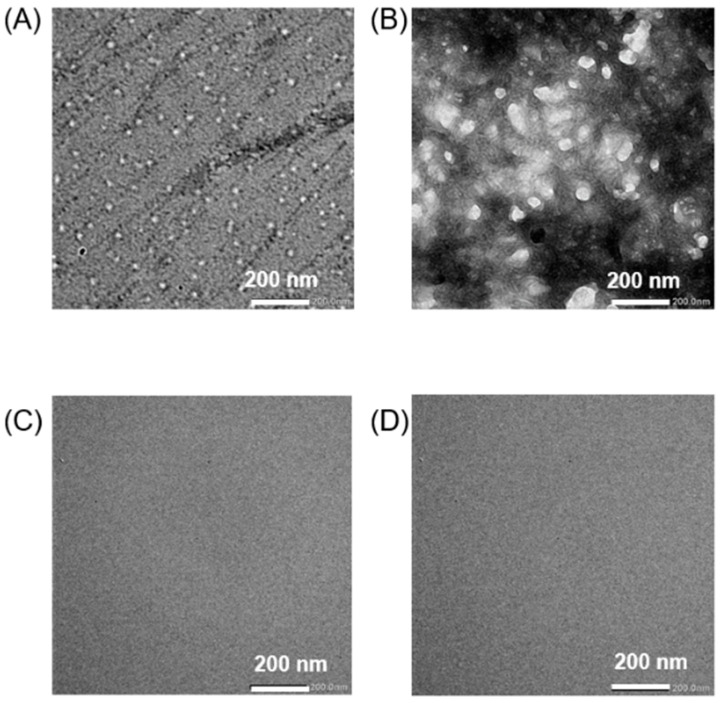
TEM images of assembly of (**A**,**C**) Chol-PEG_2000_ and (**B**,**D**) Chol-PEG_500_ at the concentration of (**A**,**B**) 470 μM above CAC and (**C**,**D**) 4.7 × 10^−5^ μM below CAC.

Cholesterol-end-modified PEGs were synthesized as reported previously [28,29]. Briefly, the synthesis was carried out by S_N_2 reaction between cholesteryl chloroformate and methoxy poly (ethylene glycol) amine (mPEG-NH_2_). Hereafter, cholesterol-end-modified PEGs using PEGs with molecular weights of 2000 and 500 are referred to as Chol-PEG_2000_ and Chol-PEG_500_, respectively. In this paper, we prepared Chol-PEG assemblies with two different concentrations, 94 µM and 470 µM, above the CAC reported previously [28,29] to study the effect of Chol-PEG concentration in Aβ_40_ inhibition experiments that follow. Figure 1 shows the particle size and zeta potential of the Chol-PEG assemblies in buffer (potassium phosphate buffer of 50 mM, pH 7.4) using dynamic light scattering (DLS) and electrophoretic light scattering (ELS). As previously reported [28,29], Chol-PEG_2000_ exhibited a size of approximately 20–30 nm in diameter, while Chol-PEG_500_ showed a size of approximately 70–80 nm in diameter at each concentration. The zeta potential was nearly neutral in both cases (Figure 1). The concentration did not affect the size and surface charge of assemblies. Figure 2A–D present the TEM images of the Chol-PEG assemblies in potassium phosphate buffer, which will be used in later Aβ experiments. Because phosphates in the buffer interacted with the staining solution, the images could not be taken clearly, so images taken using water as the solvent are shown in Figure S1. TEM images showed that Chol-PEG_2000_ formed a uniform micelle and its particle diameter was consistent with the DLS results (Figure 1 and Figure S2). On the other hand, Chol-PEG_500_ self-assembled into a uniform hollow vesicle with the uniform size of 70–80 nm, which corresponds to DLS results (Figure 1 and Figure S2). At 4.7 × 10^−5^ μM below the CAC, no particles were observed in either Chol-PEG_2000_ or Chol-PEG_500_ (Figure 2C,D). As a result, the micelles and vesicles with neutral surface charges, which have been reported to interact with Aβ [21,22], were successfully obtained.

### 3.2. ThT Assay of Aβ_40_/Chol-PEG Assemblies

**Figure 3:**
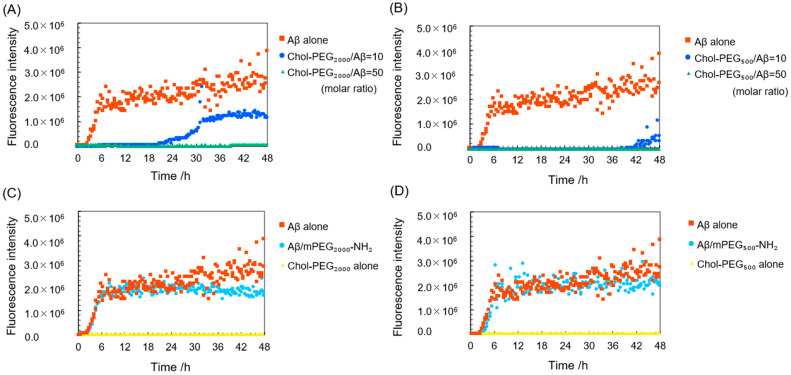
ThT assay results of Aβ_40_ aggregation in the presence of each concentration of Chol-PEGs. (**A**) Aβ_40_ incubated with Chol-PEG_2000_, (**B**) Aβ_40_ incubated with Chol-PEG_500_, (**C**) Aβ_40_ incubated with mPEG_2000_-NH_2_ and Chol-PEG_2000_ alone (without Aβ_40_), (**D**) Aβ_40_ incubated with mPEG_500_-NH_2_ and Chol-PEG_500_ alone (without Aβ_40_). The final concentration of Chol-PEGs at Chol-PEG/Aβm = 10 and Chol-PEG/Aβ = 50 is 94 µM and 470 µM, respectively.

To investigate the aggregation inhibitory effect of Chol-PEGs on Aβ, a ThT assay [36,37] was performed. ThT is a fluorescent reagent known to specifically bind to β-sheets, resulting in increased fluorescence intensity. Since Aβ forms a β-sheet structure upon aggregation, the fluorescence intensity derived from ThT increases with the aggregation of Aβ_40_. The graph showing the fluorescence intensity of each sample over time is presented in Figure 3. The figure with error bars was complicated, so it is shown in Figure S3. In the sample of Aβ_40_ alone, the fluorescence intensity began to increase at around 3 h and stabilized at around 6–7 h, confirming that aggregation was progressing. This is a typical result of a ThT assay showing Aβ aggregation [38]. When Aβ_40_ was incubated with Chol-PEG assemblies at high concentrations (50 times the molar ratio relative to Aβ_40_, 470 µM), neither Chol-PEG_2000_ micelles nor Chol-PEG_500_ vesicles showed a significant increase in fluorescence intensity throughout the measurement period. At low concentrations (10 times the molar ratio relative to Aβ_40_, 94 µM), the mixture of Aβ_40_ and Chol-PEG assemblies delayed the onset time of increase in fluorescence intensity, which indicates that Chol-PEG delayed a nucleation of Aβ [39] (Figure 3A,B). In detail, the mixture of Aβ_40_ and Chol-PEG_2000_ showed an increase of fluorescence intensity after 20 h, while the increase of fluorescence intensity was observed after 40 h in the mixture of Aβ_40_ and Chol-PEG_500_. These results indicate that Chol-PEG_500_ delayed nucleation longer than Chol-PEG_2000_. Furthermore, when low concentrations of Chol-PEG_2000_ were added, the value at which the fluorescence intensity plateaued was approximately half that of Aβ_40_ alone (Figure S4A). It has been reported that increasing fluorescence intensity of ThT means fibril elongation [40]. This suggests that fibril elongation is stopped midway. Further, an incubation up to 72 h showed that when 10 equivalents of Chol-PEG_500_ were added to Aβ_40_, the fluorescence intensity at the plateau was 30–40% lower than that of Aβ_40_ alone and lower than that of the mixture with Chol-PEG_2000_. (Figure S4B). The fluorescence intensities measured at the end of the experiment were 3.2 × 10^6^ for Aβ alone (FI_Aβ_), 1.1 × 10^6^ for the mixture with Chol-PEG_2000_ (FI_2000_), and 5.9 × 10^5^ for the mixture with Chol-PEG_500_ (FI_500_). The binding ratio of Aβ_40_ to each Chol-PEG can be estimated from (FI_Aβ_ − FI_2000_)/FI_Aβ_ and (FI_Aβ_ − FI_500_)/FI_Aβ_, assuming that Aβ_40_ not bound to Chol-PEGs forms fibrils. Applying this formula, the binding ratio of Chol-PEGs to Aβ_40_ is calculated to be 64% for Chol-PEG_2000_ [(FI_Aβ_ − FI_2000_)/FI_Aβ_ × 100] and 84% for Chol-PEG_500_ [(FI_Aβ_ − FI_500_)/FI_Aβ_ × 100], respectively. Namely, Chol-PEG_500_ exhibited 1.3 times the binding affinity for Aβ_40_ compared to Chol-PEG_2000_. These results indicate that Chol-PEG_2000_ and Chol-PEG_500_ suppressed the Aβ_40_ aggregation at a higher molar ratio than 50 and 10 against Aβ_40_, respectively. So far, some reports have shown that cholesterol promotes Aβ aggregation [41,42], but our results were the opposite, inhibiting Aβ aggregation. In the ThT assay, it is known that the period from when the fluorescence intensity begins to increase until it plateaus reflects the elongation of amyloid fibrils [40]. In this study, no significant changes in elongation rate were observed at high concentrations of Chol-PEG_2000_ and at all concentrations of Chol-PEG_500_, suggesting that Chol-PEGs might suppress nucleation before Aβ monomers form oligomers. Next, ThT assays were performed by adding mPEG-NH_2_ of two molecular weights to Aβ_40_ as controls. The fluorescence intensity with mPEG_2000_-NH_2_ was reduced by approximately –30% for PEG_2000_ compared to Aβ_40_ alone (Figure 3C). It has been reported that the presence of polymers such as PEG can inhibit the binding of Aβ to ThT [43,44]. However, such an inhibitory effect of PEG on the binding of Aβ_40_ to ThT had minimal impact on the results in our case. The mixture of Chol-PEG alone and ThT (without Aβ_40_) showed no increase in fluorescence intensity (Figure 3C,D). This means that both the cholesterol moiety and the PEG chain play an important role in inhibiting Aβ_40_ fibril formation, and the importance of Chol-PEG, a covalent conjugate of cholesterol and PEG, has been proved.

### 3.3. Evaluation of β-Sheet Formation with CD Measurement

**Figure 4:**
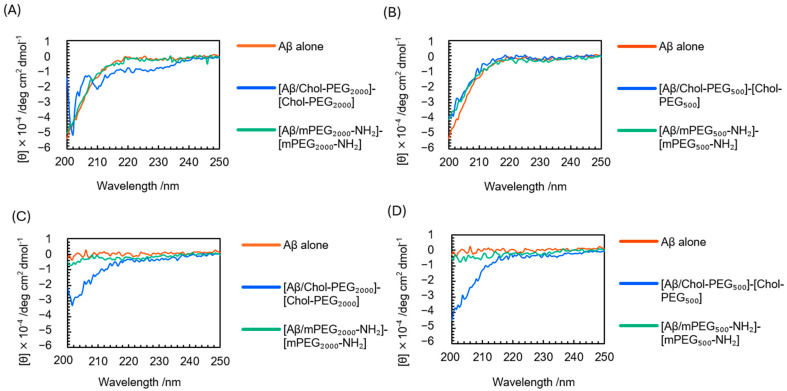
CD spectra of Aβ_40_ and Chol-PEG mixtures. (**A**,**B**) CD spectra immediately after mixing (0 h) of Aβ_40_ with (**A**) Chol-PEG_2000_ or (**B**) Chol-PEG_500_, and their respective controls, mPEG_2000_-NH_2_ and mPEG_500_-NH_2_. (**C**,**D**) CD spectra after 48 h of incubation (48 h) of Aβ_40_ with (**C**) Chol-PEG_2000_ or (**D**) Chol-PEG_500_, and their respective controls.

CD spectroscopy was performed to confirm the secondary structure of Aβ_40_ before and after incubation with Chol-PEG assemblies. Aβ_40_ was prepared at the same concentrations as in the ThT assay. Immediately after mixing Aβ_40_ and Chol-PEG assembly (0 h), Aβ_40_ exhibited a random coil conformation, and the spectrum was consistent with previously reported results [45,46] (Figure 4A,B). The Chol-PEG assemblies themselves are optically active, exhibiting a spectrum with a minimum around 205 nm (Figure S5). Therefore, to facilitate comparison, the ellipticity of Chol-PEG alone was subtracted from the CD ellipticities obtained for all Aβ_40_ and Chol-PEG mixtures, and the difference was plotted as [Aβ/Chol-PEG]–[Chol-PEG]. Each sample was incubated at 37 °C for 48 h, after which CD spectra were measured under the same conditions. After 48 h, the Aβ_40_-only sample showed an increase in the short wavelength region of 200–220 nm, which initially showed negative values at 0 h and shifted to near-zero values (Figure 4C,D). It is known that Aβ_40_ forms β-sheets from naked random coils as they aggregate, with a positive maximum around 197 nm and a cross-over at 203–206 nm [47]. In both Chol-PEG_2000_ and Chol-PEG_500_, Aβ_40_ mixed with Chol-PEGs exhibited a random coil-like spectrum similar to that of Aβ_40_ alone at 0 h. Even at 48 h, the Aβ_40_/Chol-PEG mixtures maintained a random coil-like spectrum for both Chol-PEG_2000_ and Chol-PEG_500_. In contrast, control samples of only PEG chains, or mPEG_2000_-NH_2_ or mPEG_500_-NH_2_ mixed with Aβ_40_, showed spectra indicative of a β-sheet structure similar to that of Aβ_40_ alone. These results suggest that in the presence of Chol-PEG, Aβ_40_ maintained its secondary structure as a random coil.

### 3.4. Aggregation Evaluation Using Native-PAGE

**Figure 5:**
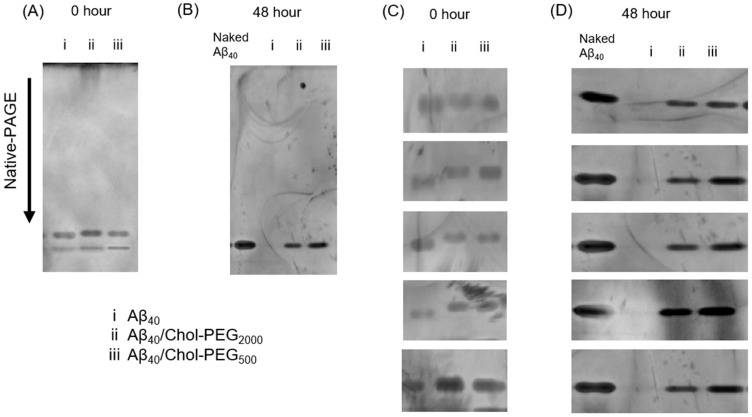
Polyacrylamide gel electrophoresis of Aβ_40_ incubated with Chol-PEGs (**A**) just after mixing Aβ_40_ and Chol-PEG assemblies, (**B**) after incubation for 48 h. (**C**) Magnified results of repeating the experiment in (**A**) five times, (**D**) magnified results of repeating the experiment in (**B**) five times.

The aggregation inhibition effect of Chol-PEG assemblies and the formation of complexes between Aβ_40_ and Chol-PEG assemblies were evaluated using polyacrylamide gel electrophoresis (Native-PAGE). As shown in Figure 5A, in the presence of Chol-PEG assemblies, Aβ_40_ led to a noticeable retardation compared to the migration profile of Aβ_40_ alone. The retardation means an increase in molecular weight. Results from multiple repeated experiments are presented in Figure 5C for clarity. In these images, the retardation reproduced in the case of mixture of Aβ_40_ and Chol-PEG assemblies. These retardations reflect the binding of Chol-PEG assemblies to Aβ_40_, leading to the formation of complexes. Electrophoresis results after 48 h of incubation under aggregation conditions are shown in Figure 5B. An incubation of Aβ_40_ alone resulted in no band which means insolubilization by aggregation (lane i). Notably, the bands of Aβ_40_ mixed with Chol-PEG assemblies appeared at the same position (no retardation) as the naked Aβ_40_ after the 48 h incubation. While retardation was observed immediately after mixing (Figure 5A), no retardation after 48 h of incubation indicates a complex dissociation for inhibition of Aβ_40_ aggregation. For more detailed study, Native-PAGE was performed after 5 min of incubation (Figure S6). Interestingly, no retardation was observed even after 5 min.

The mixture of mPEG-NH_2_ and Aβ_40_ showed a band identical to that of Aβ_40_ alone (Figure S7A), which disappeared following incubation (Figure S7B). Additionally, no bands were observed in individual polymers alone (lanes iv–vii).

### 3.5. Disaggregation Effect of Chol-PEG Assembly on Aggregated Aβ

Finally, the disaggregation effect of Chol-PEG_500_ vesicles was evaluated using the ThT assay by addition of 50 equivalents of Chol-PEG_500_ vesicles to aggregated Aβ_40_ (Figure S8). Aggregated Aβ_40_ was prepared by incubating at 37 °C for 72 h using the same method as the ThT assay in Section 3.2. The fluorescence intensity was maintained during incubation for 3 days, which indicates that Aβ_40_ kept aggregation form and was not disaggregated by adding Chol-PEG_500_ vesicles.

## 4. Discussion

**Figure 6:**
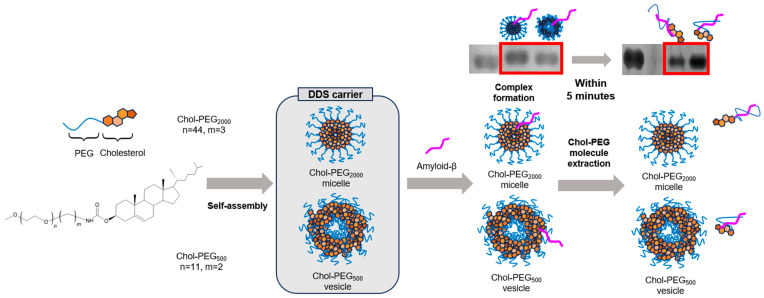
Hypothesis on the mechanism by which Chol-PEG inhibits Aβ_40_ aggregation.

Native-PAGE results showed that retardation was observed immediately after mixing Aβ_40_ and Chol-PEG assemblies, but not after 48 h of incubation. Although no retardation was observed after 48 h, it is unlikely that the complexes completely dissociate from each other because the ThT assay and CD spectra showed that the aggregation was inhibited from 0 h to 48 h. For a more detailed study of the mechanism, Native-PAGE was performed 5 min after mixing (Figure S6). The results showed that no retardation was observed even after 5 min. Although it is difficult to perform Native-PAGE at shorter time points due to the experimental manipulations, at least within 5 min, the complexes of Aβ_40_ and Chol-PEG micelles or vesicles are dissociated. According to a report by Hashemi M et al., aggregation of Aβ bound to lipid bilayers was detected after 1 h. Since binding to Chol-PEG assemblies was resolved within 5 min, it is suggested that aggregation was not enhanced [41]. If the complex formation had completely dissociated, it would not be possible to explain the suppression of aggregation over a 48 h period. Therefore, we propose a mechanism by which Aβ_40_ pulls out Chol-PEG molecules from Chol-PEG micelles and vesicles (Figure 6).

PEGs can remove hydration from biopharmaceuticals and is used as a precipitant. Therefore, as the molecular weight of PEG increases, the dehydration of peptides intensifies, potentially triggering aggregation [48,49]. Moreover, it has been reported that a higher PEG molecular weight leads to increased steric hindrance, reducing interactions between the hydrophobic group and the peptide [50]. The surface of Chol-PEG assemblies is covered by hydrophilic PEG chains, and it was considered that Aβ_40_ was more likely to interact with cholesterol in Chol-PEG_500_, which has shorter PEG chains. Therefore, in this study, it is considered that Chol-PEG_500_, with its shorter PEG chains, inhibited aggregation more effectively compared to Chol-PEG_2000_. These binding and aggregation inhibitory effects in this study were contributed by several interactions reported in the literature [48,49,51,52,53,54,55,56].

In addition, we evaluated whether the addition of 50 equivalents of Chol-PEG_500_ to aggregated Aβ_40_ would cause disaggregation using the ThT assay. As a result, no decrease in fluorescence intensity was observed, and it was found that there was no disaggregation effect on already aggregated Aβ_40_ (Figure S8). When Aβ_40_ aggregates, hydrophobic amino acids (e.g., Phe-19, Phe-20, etc.) that bind to cholesterol in the vesicle contribute to the formation of the core and are hidden inside [57,58]. Therefore, the interaction with cholesterol-containing vesicles consisting of phospholipids is known to be reduced, as compared to that with naked Aβ. This suggests that Aβ_40_ and Chol-PEGs are bound to hydrophobic amino acids that are known to easily bind to cholesterol. It is well known that vesicles containing free (non-covalently bound) cholesterol and Aβ bind to each other, accelerating aggregation [59,60]. In this study, Chol-PEG is a covalently bonded cholesterol and PEG, and even after the dissociation complex between Aβ and Chol-PEG, the binding to the amphiphilic Chol-PEG molecules remained, so it is thought that aggregation was suppressed. While Aβ has been a primary focus in this research, other factors, such as tau protein and APOEε4, among others, are also known to contribute to its pathogenesis [61]. As previously reported, Chol-PEG assemblies can encapsulate therapeutic agents [28,29]. Therefore, by encapsulating additional AD-related factors, these assemblies offer potential for dual-targeted AD therapy via intranasal administration.

## 5. Conclusions

In this study, we found that the vesicle and micelle composed of Chol-PEG had aggregation-inhibiting effects on Aβ_40_, the causative agent of AD. These Chol-PEG assemblies with a high affinity for Aβ_40_ result in the absorption of Aβ_40_ into the Chol-PEG assemblies and suppress the aggregation of Aβ_40_. The reasons for this were expected to be that the surface PEG layer of Chol-PEG_500_ vesicles was thinner than that of Chol-PEG_2000_ micelles, making Aβ_40_ more likely to interact with cholesterol, and the too-long PEG chain length of Chol-PEG_2000_ causes dehydration [50] of Aβ_40_, inducing Aβ_40_ aggregation. Furthermore, the suppression of aggregation was considered to be due to hydrogen bonding with the urethane group of Chol-PEGs, the exclusion volume effect of PEG, and its high hydrophilicity The resulting suppression effect of Chol-PEG assemblies on Aβ_40_ aggregation is promoted at higher concentrations of the dispersion of the Chol-PEG assemblies. Although general cholesterol-containing micelles [20] and vesicles consisting of phospholipids are known to promote Aβ aggregation [51,52], our Chol-PEG assemblies used in this study proved to have moderate binding strength that rather inhibited aggregation. This provides an idea for the development of therapeutic agents in the study of interaction between Aβ and vesicles. Moreover, Chol-PEG_500_ vesicles, as well as Chol-PEG_2000_ micelles, can encapsulate hydrophilic and hydrophobic drugs due to their hollow shape with internal water phase and hydrophobic membrane. These Chol-PEG assemblies are designed to both inhibit Aβ aggregation and simultaneously deliver additional therapeutic agents via intranasal administration, offering a dual-functional approach for effective AD treatment.

## Data Availability

All the data supporting the reported results can be found in the main article and in the Supplementary Materials files.

## Supplementary Materials

The following supporting information can be downloaded at: https://www.mdpi.com/article/10.3390/pharmaceutics17010001/s1, Figure S1: TEM image of Chol-PEG_500_ aqueous solution; Figure S2: Particle sizes of Chol-PEG assemblies measured from TEM images; Figure S3: ThT assay results of Aβ_40_ aggregation in the presence of each concentration of Chol-PEGs with error bars; Figure S4: ThT assay results of Aβ_40_ aggregation in the presence of each concentration of Chol-PEGs for 72 h with error bars; Figure S5: CD spectra of Chol-PEG alone; Figure S6: Polyacrylamide gel electrophoresis of Aβ_40_ incubated with Chol-PEGs 5 min after mixing Aβ and Chol-PEG assemblies; Figure S7: Polyacrylamide gel electrophoresis of each polymer incubated with or without Aβ_40_ as controls; Figure S8: Results of the ThT assay for aggregated Aβ_40_.
