# Supplementary material for: Inhibition of Aβ Aggregation by Cholesterol-End-Modified PEG Vesicles and Micelles
Source: Pharmaceutics. 2024 Dec 24;17(1):1. doi: 10.3390/pharmaceutics17010001 (PMC11769297; doi:10.3390/pharmaceutics17010001)
Supplement: Supplementary file 1 [file pharmaceutics-17-00001-s001.zip › pharmaceutics-3356153-supplementary.pdf]

Supplementary Materials

Inhibition of Aβ aggregation by cholesterol-end-modified PEGs vesicle and micelle

Shota Watanabe, Motoki Ueda and Shoichiro Asayama*

Department of Applied Chemistry, Tokyo Metropolitan University, Hachioji, Tokyo 192-0397, Japan

* Email: asayama-shoichiro@tmu.ac.jp

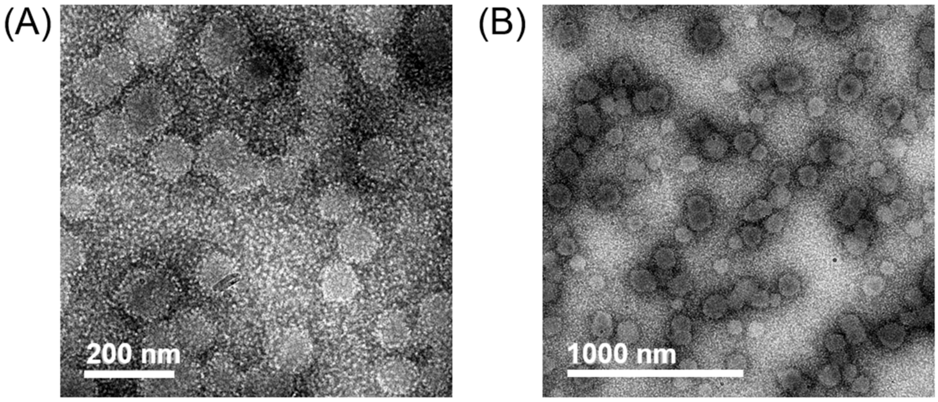

Figure S1. TEM image of 5.5 mM (above CAC) aqueous solution of Chol-PEG_{500} vesicles (above CAC).

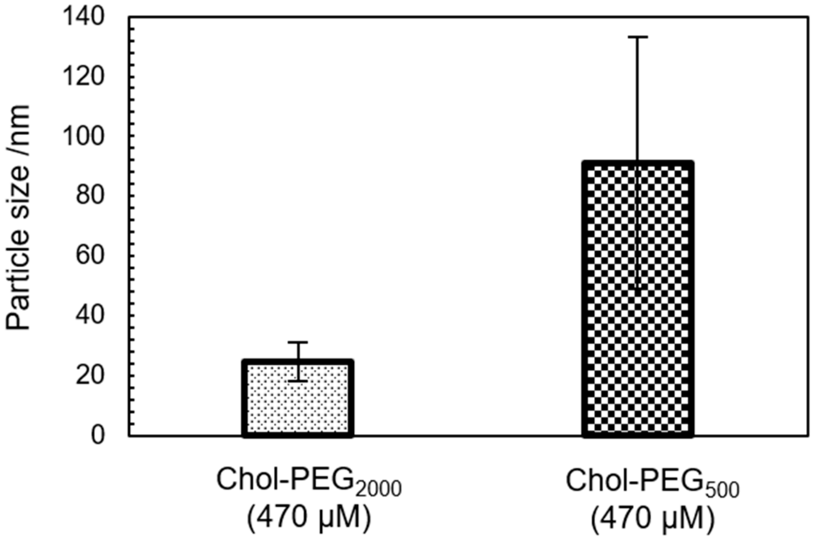

Figure S2. Particle size measured from TEM images: n=169 for Chol-PEG_{2000}, n=92 for Chol-PEG_{500}.

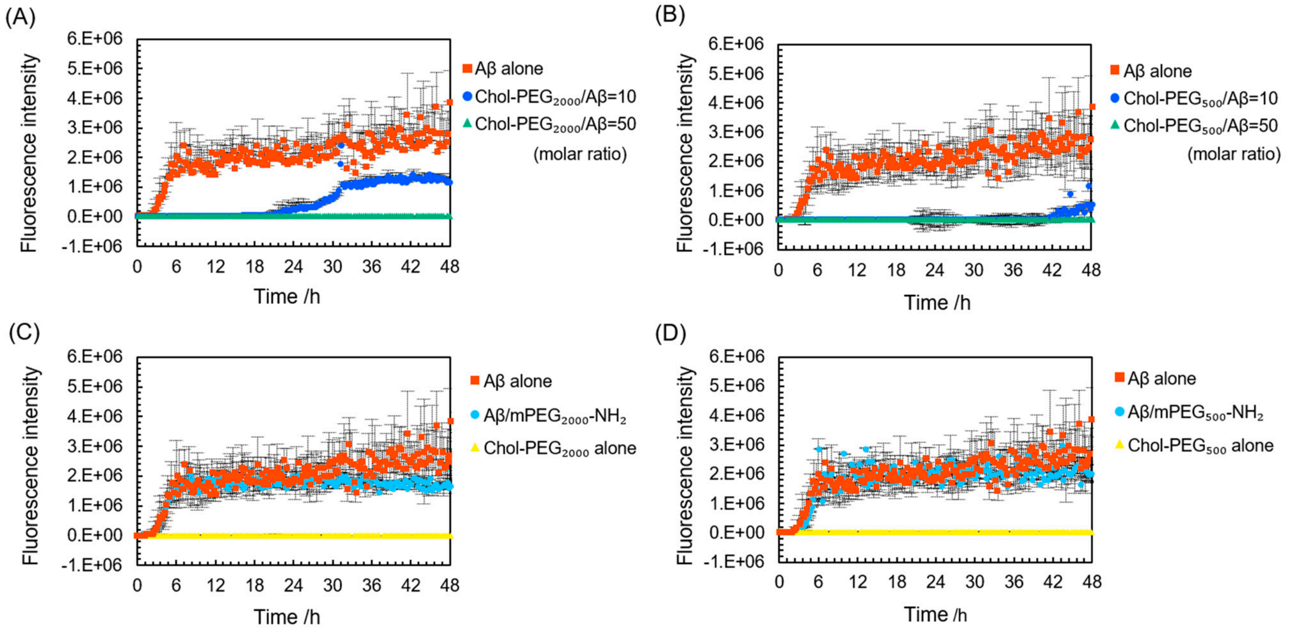

Figure. S3. ThT assay results of $A\beta_{40}$ aggregation in the presence of each concentration of Chol-PEGs with error bars. (A) $A\beta_{40}$ incubated with Chol-PEG$_{2000}$, (B) $A\beta_{40}$ incubated with Chol-PEG$_{500}$, (C) $A\beta_{40}$ incubated with mPEG$_{2000}$-NH_2 and Chol-PEG$_{2000}$ alone (without $A\beta_{40}$), (D) $A\beta_{40}$ incubated with mPEG$_{500}$-NH_2 and Chol-PEG$_{500}$ alone (without $A\beta_{40}$). The final concentration of Chol-PEGs at Chol-PEG/Aβ=10 and Chol-PEG/Aβ=50 is 94 μM and 470 μM, respectively.

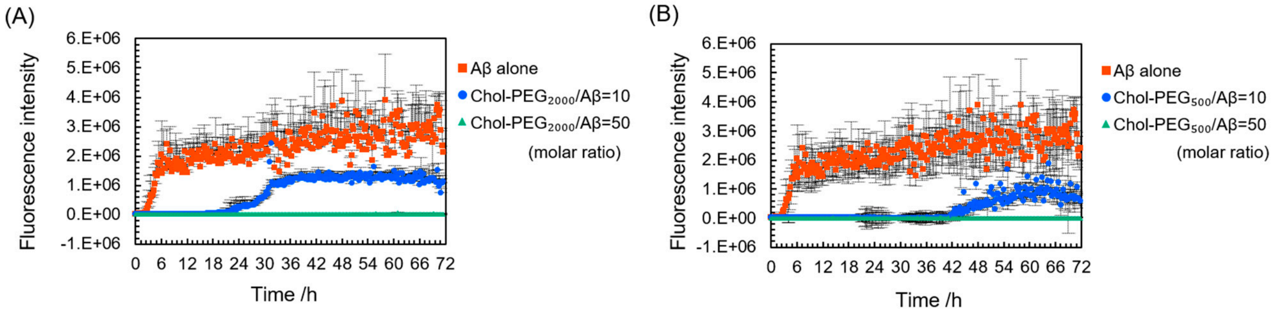

Figure.S4. ThT assay results of $A\beta_{40}$ aggregation in the presence of each concentration of Chol-PEGs with error bars. (A) $A\beta_{40}$ incubated with Chol-PEG_{2000}, Chol-PEG/$A\beta_{40}$ (B) $A\beta_{40}$ incubated with Chol-PEG_{500}. The final concentration of Chol-PEGs at Chol-PEG/Aβ=10 and Chol-PEG/Aβ=50 is 94 μM and 470 μM respectively.

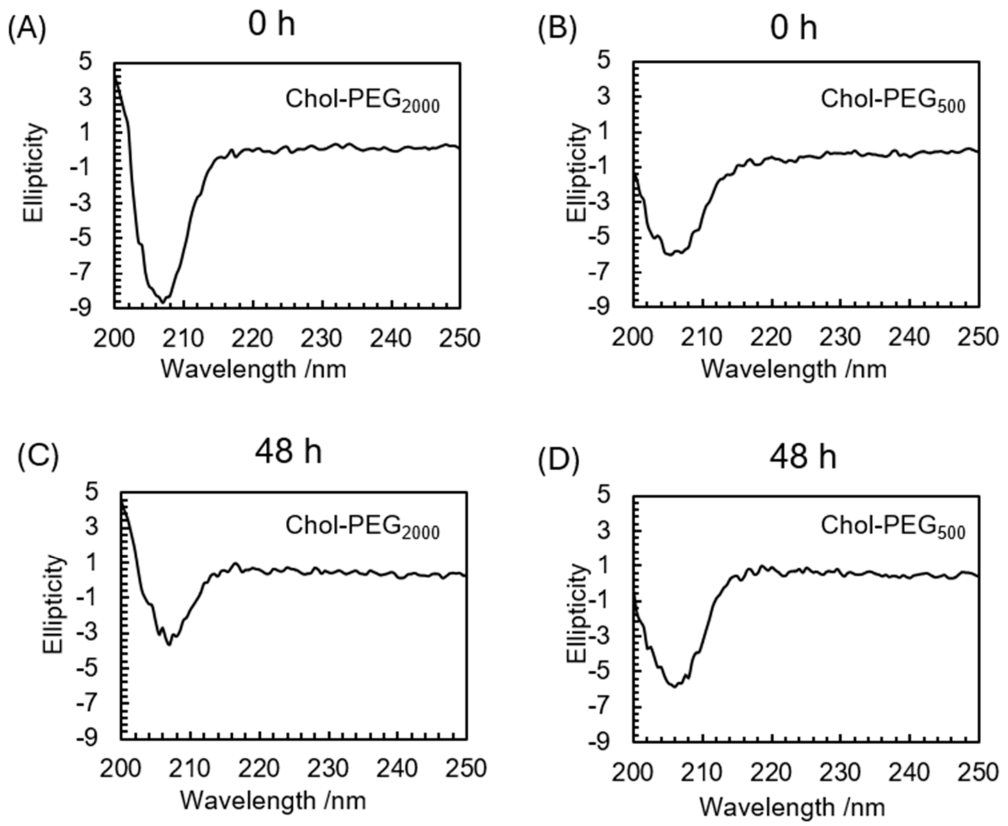

Figure.S5. CD spectra of Chol-PEG alone. (A) Immediately after dissolving Chol-PEG_{2000}, (B) immediately after dissolving Chol-PEG_{500}, (C) after incubating Chol-PEG_{2000} for 48 hours, (D) after incubating Chol-PEG_{500} for 48 hours.

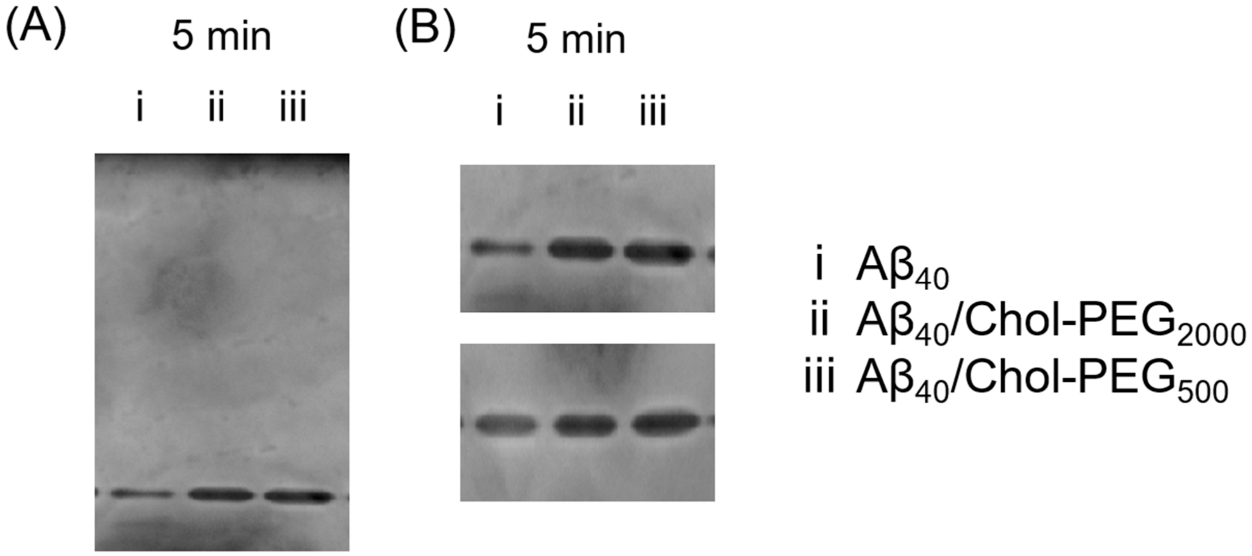

Figure S6. Polyacrylamide gel electrophoresis of $A\beta_{40}$ incubated with Chol-PEGs: (A) 5 minutes after mixing $A\beta_{40}$ and Chol-PEG assemblies and (B) As each magnified result of repeating the experiment two times.

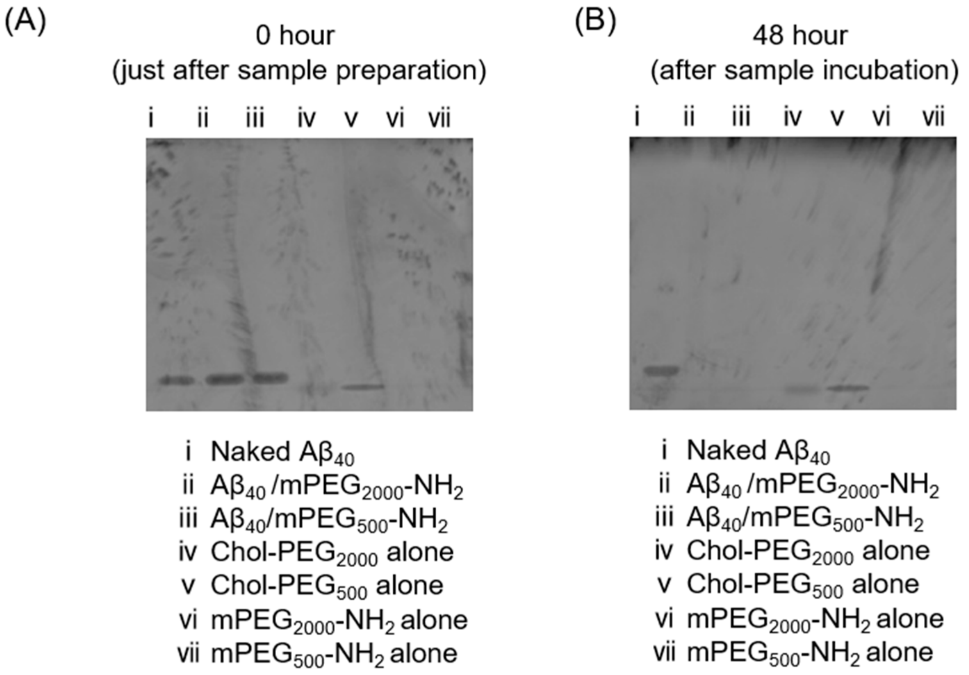

Figure.S7. Polyacrylamide gel electrophoresis of each polymer incubate with or without $A\beta_{40}$. (A) Immediately after sample preparation, (B) After incubate 48 hour.

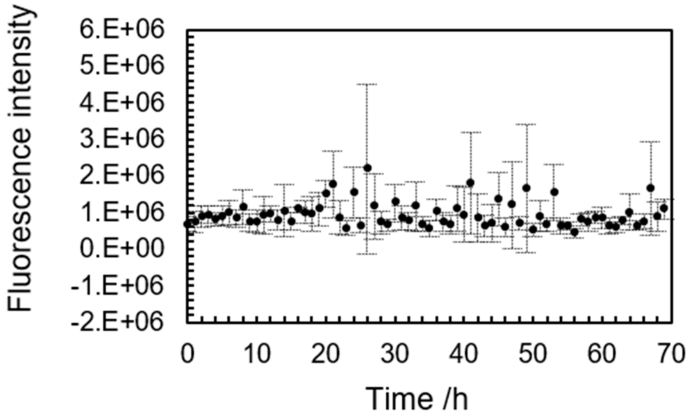

Figure S8. Results of the ThT assay for Aβ_{40}. Fluorescence intensity changes over time after adding 50 equivalents of Chol-PEG$_{500}$ by molar ratio after incubating Aβ_{40} for 72 hours.
